# Short-term effect of *Eucalyptus* plantations on soil microbial communities and soil-atmosphere methane and nitrous oxide exchange

**DOI:** 10.1038/s41598-018-33594-6

**Published:** 2018-10-11

**Authors:** Caroline A. Cuer, Renato de A. R. Rodrigues, Fabiano C. Balieiro, Jacqueline Jesus, Elderson P. Silva, Bruno José R. Alves, Caio T. C. C. Rachid

**Affiliations:** 1 LABEM - Laboratory of Biotechnology and Microbial Ecology - Institute of Microbiology Paulo de Góes, Federal University of Rio de Janeiro, Rio de Janeiro, Brazil; 2 University of Strasbourg, Strasbourg, France; 3 Secretary of Intelligence and Strategic Affairs - Embrapa, Brasilia, Brazil; 4 Embrapa Soils, Rio de Janeiro, Brazil; 5 Fluminense Federal University, Rio de Janeiro, Brazil; 6 Embrapa Agrobiologia, Seropédica, Brazil

## Abstract

Soil greenhouse gas (GHG) emissions are a significant environmental problem resulting from microbially-mediated nitrogen (N) and carbon (C) cycling. This study aimed to investigate the impact of *Eucalyptus* plantations on the structure and function of a soil microbial community, and how resulting alterations may be linked to GHG fluxes. We sampled and monitored two adjacent *Eucalyptus* plantations—a recently logged site that harbored new seedlings and an adult plantation—and compared them to a site hosting native vegetation. We used 16S rRNA gene sequencing and qPCR amplifications of key nitrogen and methane cycle genes to characterize microbial structure and functional gene abundance and compared our data with soil parameters and GHG fluxes. Both microbial community attributes were significantly affected by land use and logging of *Eucalyptus* plantations. The genes *nosZ* and archaeal *amoA* were significantly more abundant in native forest than in either young or old *Eucalyptus* plantations. Statistical analyses suggest that land use type has a greater impact on microbial community structure and functional gene abundance than *Eucalyptus* rotation. There was no correlation between GHG fluxes and shifts in microbial community, suggesting that microbial community structure and functional gene abundance are not the main drivers of GHG fluxes in this system.

## Introduction

Emissions of greenhouse gases (GHG) through human activities represent a pressing issue today, contributing to global climate change and ecosystem destabilization^1^. Atmospheric concentrations of the three main GHGs—carbon dioxide (CO_2_), nitrous oxide (N_2_O) and methane (CH_4_)—have increased by 144%, 256% and 121%, respectively, since the first Industrial Revolution^2^. Although N_2_O and CH_4_ are less concentrated in the atmosphere than CO_2_, they represent major GHGs as their global warming potentials are respectively 296 and 25 times higher than CO_2_ over a 100-year period^3^.

Agriculture, forestry and other land uses are the third-highest sources of anthropogenic GHG emissions (24% of total GHG emissions^4^, mainly through crop cultivation and tropical deforestation. Owing to high levels of deforestation, land use change, and improper land use practices, Brazil has been ranked as the fourth-highest emitter of GHGs in the world^5^.

Planted forests cover 7.8 million ha in Brazil^6^, and they are thought to play many positive roles in the context of climate change and deforestation through restoration of degraded land, soil conservation, CO_2_ sequestration, and protection of biodiversity. Their appropriate use in many industrial applications also reduce pressures on native forests^7^.

Of these Brazilian planted forests, 5.56 million ha are dedicated to *Eucalyptus*. *Eucalyptus* are fast-growing trees with high carbon sequestration potential during development^8,9^. *Eucalyptus* plantations have been reported to be a source of N_2_O and CO_2_ and a sink of CH_4_ in semi-arid and subtropical climates, as observed for most forest ecosystems^10,11^. However, GHG fluxes in *Eucalyptu*s plantations have not yet been well described in the tropics, so a greater understanding of the impacts of *Eucalyptu*s plantation management on these fluxes is still needed.

Soil behaves as both source and sink for GHGs^12^, as it represents the living space for the microbial communities responsible for nutrient cycling^13^. Accordingly, microbial activities in the N and C cycles are central to GHG fluxes in soil.

The link between soil microbial communities and GHG fluxes has previously been described^14^. Soil microbial processes are particularly impacted by land use practices, which can deregulate nutrient cycles and thereby increase or reduce GHG emissions^15,16^. Some studies have revealed a correlation between the abundance and/or expression of functional genes involved in N and C cycles and GHG fluxes in forest soils^16–18^. However, until now, no study has focused on the link between a microbial community and the GHG fluxes in the soil of *Eucalyptus* plantations.

To address this topic, we studied GHG fluxes and the microbial community associated with *Eucalyptus* plantations at two growth stages (i.e., one with new seedlings and one with 6-year-old trees), and with a native Brazilian tropical forest (Atlantic Forest). We hypothesized that: (1) replacement of native vegetation by *Eucalyptus* plantation or *Eucalyptus* plantation rotation would lead to changes in microbial community structure and functional gene abundance; and (2) changes in microbial community would alter GHG flux dynamics at each site.

We employed two different experimental strategies to test these hypotheses. Firstly, we performed 16S ribosomal RNA (rRNA) gene sequencing to compare the composition of soil microbial communities among the three treatments (i.e., young and old *Eucalyptus* plantations, as well as native forest). Secondly, we measured by quantitative PCR (qPCR) the abundances of the following key functional genes involved in the methane and N cycles: *nifH* (nitrogen fixation), archaeal and bacterial *amoA* (nitrification), *nirK* (denitrification), *nosZ* (N_2_O reduction) and *mcrA* (CH_4_ production). We then compared our 16S rRNA sequencing and qPCR data with soil physicochemical properties and measurements of GHG fluxes.

## Materials and Methods

### Field site description

The experimental field site is located in Belo Oriente, Minas Gerais, Brazil (18–20°S, 42–44°W, 300 m elevation), in an area that belongs to the Celulose Nippo-Brasileira (CENIBRA) company. The climate is classified as Aw (tropical with a dry winter and a rainy season during summer, Köppen classification), with an annual mean temperature varying from 22 °C to 27 °C (maximum = 32 °C, minimum = 18 °C). Annual mean precipitation varies from 701 to 1,500 mm. The soil is classified as loamy red-yellow Ferralsol. The experimental area is highly sloping, with a slope of 26 degrees.

The experimental field was originally covered by Atlantic Forest, a native tropical forest. Since 1960, CENIBRA has managed *Eucalyptus* plantations in this area and adopts regular rotation cycles of 7 to 9 years between planting seedlings and tree-cutting. Part of the area has been retained as native vegetation, in accordance with Brazilian law, which allowed us to compare adjacent areas covered by native forest or *Eucalyptus* plantation. Planted seedlings are clones of *Eucalyptus urograndis* produced by the company.

We chose an area under *Eucalyptus* plantation since 1978, immediately adjacent to a fragment of Atlantic Forest to perform this study. The most recent *Eucalyptus* rotation started in 2011, with trees in the 6^th^ year growth in the beginning of 2017. To understand the short-term impact of a new rotation, we manually logged approximately half of this 6-year-old *Eucalyptus* plantation in February 2017 and replanted it immediately with new *Eucalyptus* seedlings. Sampling for our analyses was conducted at the end of March 2017.

### Experimental Design

Three adjacent areas under contrasting use were considered for this study:

i. Atlantic Forest fragment (NF) – native Brazilian tropical forest fragment (Atlantic Forest);
ii. Old *Eucalyptus* (OE) - *Eucalyptus* plantation with 6-year-old trees;
iii. Young *Eucalyptus* (YE) *- Eucalyptus* plantation in which 6-year-old trees had been removed from the field, and new seedlings were planted one month before sampling.

We compared the *Eucalyptus* plantations at the beginning (YE) and at the end of the rotation cycle (OE), with the Atlantic Forest (NF) fragment acting as a reference. OE and YE areas comprised 470 and 680 *Eucalyptus* trees, respectively, which had been planted in lines with a spacing of 3 × 2.5 m.

### GHG sampling

Gas sampling was done daily, during four days, from 14 to 17 March 2017. Nitrous oxide and methane fluxes were manually quantified using closed static chambers, similar to those described by Alves *et al*.^19^. The top-base chambers had a base composed of a rectangular steel frame (40 cm × 60 cm), which was deployed between rows of *Eucalyptus* trees or randomly in the Atlantic Forest fragment. The base was inserted into the soil to a depth of 6–7 cm, before attaching a polyethylene lid (of the same lateral dimensions as the base) to it, generating an internal chamber space 12–15 cm above the soil surface. Soft rubber was fixed to the rim of the lid to satisfactorily seal it to the base. Air accumulating in the chamber headspace was withdrawn through a three-way valve connected to the lid using a polypropylene syringe. Approximately 30 mL of this air was sampled and transferred to 20 mL chromatography vials crimped with chlorobutyl septa. Just before gas transfer, each vial was evacuated to approximately −100 kPa by using an electric vacuum pumping system. The chamber lid was covered with a 2-cm-thick foam layer and reflective adherent mantle for thermic insulation. The bases of the chambers remained in their respective areas throughout the monitoring period. After chamber closure an air sample was immediately taken from chamber headspace. Subsequent samples were taken at 20, 40 and 60 min, after which lids were removed. Gas sampling was always performed in the morning between 08 h and 10 h^19^.

Gas analyses were performed using a gas chromatograph (GC 2014, Shimadzu, Japan). For each sampling run, N_2_O and CH_4_ standards were used to build an analytical curve to transform the integrated areas of each sample peak into gas concentrations.

Gas fluxes were calculated by the equation F = (ΔC/Δt)(V/A)M/Vm, where ΔC/Δt is the slope of a linear function fitted to the gas concentration of samples taken at 0, 20, 40 and 60 min after chamber closure; V and A are the volume of the chamber and the area of soil covered by the chamber, respectively; M is the molecular weight of atoms of the elements N and C, respectively, in molecules of N_2_O and CH_4_; and Vm is the molecular volume at the sampling temperature.

### Biogeochemical characteristics of the soil

We sampled approximately 500 g of soil from nearby each of the gas chambers described in the previous section (around 10 cm from the chamber) to measure clay content, pH, total carbon, total nitrogen, available P, and amounts of H^+^ + Al^3+^, Ca^2+^, Mg^2+^ and Al^3+^. Each sampling point served as an independent replicate, resulting in five replicates per treatment, which were used to assess correlations with the respective gas fluxes calculated for each chamber.

Soil samples were air dried, sieved (2 mm) and analysed chemically. Total carbon and total nitrogen were determined using a CHN elemental analyser (PerkinElmer, USA). Exchangeable nutrients: Ca^2+^, Mg^2+^ and Al^3+^ were extracted by 1 M KCl; P, Na and K by Mehlich-1 extractant −0.05 mol L^−1^ in HCl in 0,0125 mol L^−1^ H_2_SO_4_) and pH (soil:water, 1:10); Potential acidity: H + Al extracted with calcium acetate 1 N (pH 7), titrated with 0.0125 NaOH N. Inductively coupled plasma apparatus for Ca^+2^, Mg^+2^ and Al^+^, flame emission (K and Na) and photocolorimeter (for P) were used to nutrient determinations. Soil granulometry was determined using the aerometer method, after chemical dispersion. All these soil characteristics were measured according to Embrapa^20^.

Soil moisture and concentrations of NO_3_^−^ and NH_4_^+^ were measured according to Morais *et al*.^21^. Determinations were made from samples taken from another four randomly selected points in each of the three areas. The inorganic N content was extracted from fresh soil with 60 mL 2M KCl after 1 h on a rotary shaker at 220 rpm. The supernatant was filtered and the NO_3_^−^ and NH_4_^+^ concentrations were determined in the resultant solution respectively by flow injection (FIA) technique using Cd reduction and nitrite analysis and by the salicylate reaction adapted for FIA. We used averages of the four values obtained for soil moisture and NO_3_^−^ and NH_4_^+^ concentrations.

### Soil sampling for microbial analyses

Soil samples were taken from five points in each treatment area (YE, OE and NF), approximately 10 cm away from each gas flux chamber, resulting in five replicates per treatment. To extract each soil sample, we placed a steel tube probe of 1.5 cm diameter (previously sterilized at 180 °C for 3 h to remove contaminants and nucleases) into the soil to a depth of 7 cm. The harvested soil was immediately put in a sterile 50 mL propylene tube for mixing, before being separated into two subsamples and placed in liquid nitrogen until we conducted DNA extractions.

### Analysis of bacterial community structure

DNA was extracted from approximately 500 mg of each soil sample using the Fast DNA Spin Kit for Soil (MP Biomedicals, USA). The extracted DNA was dark in color due to a high level of humic material, so it was then purified using the last steps of the NucleoSpin Soil kit (Macherey-Nagel, Germany) protocol. DNA concentration and quality were measured using a NanoDrop (Thermo Fisher, USA). Soil bacterial diversity were assessed by next generation sequencing of the V4 variable region of the 16S rRNA gene using the primers 515FB (GTGYCAGCMGCCGCGGTAA) and 806RB (GGACTACNVGGGTWTCTAAT)^22,23^.

PCR reactions with a barcode on the forward primer were used in a 28-cycle PCR using the HotStarTaq Plus Master Mix Kit (Qiagen, USA) under the following conditions: 94 °C 3 min; 28 cycles of (94 °C 30 s, 53 °C 40 s, 72 °C 1 min); 72 °C 5 min. Following PCR amplification, PCR products were checked in 2% agarose gels to determine the success of PCR and the relative intensity of resulting bands. Multiple samples were pooled in equal proportions based on their molecular weight and DNA concentrations. Pooled samples were purified using calibrated Ampure XP beads (Beckman Coulter, USA). The pooled and purified PCR products were then used to prepare an Illumina DNA library.

Sequencing was performed by MR DNA (www.mrdnalab.com, Shallowater, TX, USA) on a MiSeq (Illumina, USA) paired-end 2 × 250 sequencing system, following the manufacturer’s guidelines. Raw data were downloaded from Basespace and analysed using Mothur v1.39 software^24^. Forward and reverse paired sequences were merged into contigs after checking for the presence of barcodes and primers in the sequences. Merged sequences of less than 240 base pairs (bp) or greater than 300 bp, containing any ambiguities, or containing more than 8-mer homopolymers were removed.

Sequences were then aligned using a modified Silva database (generated by a virtual PCR using the same primers as those used for our samples) as reference^25^, and the resulting alignment was submitted to screen.seqs and filter.seqs (Mothur v1.39) to remove badly aligned sequences and uninformative columns in the alignment. The sequences where then pre-clustered using the command pre.cluster (Mothur v1.39.5) with parameter diffs = 2. Chimeras were detected using the command chimera.vsearch (Mothur v1.39.5) and then eliminated. We classified sequences using the classify.seqs (Mothur v1.39.5) command, with the Ribosomal Database Project^26^ as reference and a bootstrap threshold of 80. Sequences identified as being from chloroplasts, mitochondria, Eukarya or Archaea and those not assigned to any kingdom were removed. The resulting sequences were used as input for the dist.seqs (Mothur v1.39.5) command.

Finally, we clustered the sequences into operational taxonomic units (OTUs), with a 3% dissimilarity threshold. To avoid bias due to sampling effort, the samples were randomly normalized to the same number of sequences (35028). We employed a taxonomic summary to assess the bacterial composition of each sample.

Differences in the relative frequencies of phyla and classes among treatments were tested using analysis of variance (ANOVA) followed by Tukey’s test. Before performing ANOVA, we checked the homoscedasticity among treatments and normality of distributions (Shapiro-Wilk’s test) for all values, and data was transformed (log or Box-Cox transformation) accordingly if necessary before performing ANOVA. The Shannon index was also analysed by ANOVA followed by a pairwise Tukey’s test.

The distribution of OTUs was used as input for a non-metric multidimensional scaling (NMDS) ordination with the Bray-Curtis similarity index to assess relationships among samples. We performed a PERMANOVA test, followed by Bonferroni correction (p < 0.05), to assess differences in structural composition among treatments. All aforementioned statistical analyses were conducted in Past3^27^. To determine if the treatments had a significant effect on specific bacterial OTUs, we undertook a blocked Indicator Species Analysis (ISA) (YE vs OE and YE + OE vs NF) using Mothur v1.39.5. The ISA, proposed by Dufrêne and Legendre^28^, is based on the relative frequency of a specie (OTU in our case) within and inter treatments. It gives provides an indicator value ranging from 100 (perfect indicator) to 0 (no indicator). The perfect indicator species would be the one found in high abundance in all samples of a given treatment and absent in all other treatments. It also uses a randomization test to evaluate the significance of the specie distribution.

### Nucleotide sequence accession numbers

The data generated were deposited in the NCBI Sequence Read Archive (SRA) and are available under Bioproject accession numbers PRJNA471919.

### Gene quantification by qPCR

Genes were chosen based on their involvement in the soil nitrogen and methane cycles. Primers were selected according to the literature and assessed with the Primer blast tool of the National Center of Biotechnology Information (NCBI) (https://www.ncbi.nlm.nih.gov/tools/primer-blast/). We chose primers with the highest number of results and lacking non-specificity (Table 1).

**Table 1:** Primers and protocols used for PCR and qPCR of targeted genes.

Target gene	Primer names	Forward primer sequence	Reverse primer sequence	Fragment size (bp)	Annealing temperature	References
16S rRNA	341 f/534r	5′-CCTACGGGAGG CAGCAG-3′	5′-ATTACCGC GGCTGCTGG-3′	193	53 °C	^51, 52^
*nifH*	PolF/PolR	5′-TGCGAYCCSA ARGCBGACTC-3′	5′ATSGCCATCATYT CRCCGGA-3′	360	55 °C	^53^
Archaeal *amoA*	19 F/CrenamoA616r48x	5′-ATGGTCTGGCT WAGACG-3′	5′-GCCATCCABC KRTANGTCCA-3′	624	55 °C	^54, 55^
Bacterial *amoA*	amoA1F/amoA2R	5′-GGGGTTTCTAC TGGTGGT-3′	5′-CCCCTCKGSA AAGCCTTCTTC-3′	491	55 °C	^56^
*nirK*	F1aCu/R3Cu	5′-ATCATGGTS CTGCCGCG-3′	5′-GCCTCGATCA GRTTGTGGTT-3′	473	62 °C	^57, 58^
*nosZ*	nosZ1F/nosZ1R	5′-WCSYTGTTCMT CGACAGCCAG-3′	5′-ATGTCGATCA RCTGVKCRTTYTC-3′	259	62 °C	^42^
*mcrA*	qmcrAf/qmcrAr	5′-TTCGGTGGAT CDCARAGRGC-3′	5′-GBARGTCGWA WCCGTAGAATCC-3′	140	58 °C	^59, 60^

The standards were constructed by amplifying each gene from DNA extracted from soil or activated sludge, ligating them into plasmids (CloneJET PCR Cloning Kit, Thermo Fisher Scientific, USA), and transforming them into *E. coli* DH5alpha plasmid (heat shock method, Froger and Hall, 2007). The plasmids were recovered using the PureYield Plasmid Miniprep system (Promega, USA). Based on the size of each gene, the weight of one nucleotide, and the plasmid concentration, we generated ten-fold serial dilutions in RNase- and DNase-free water for each plasmid to reach 10^10^ to 10^2^ gene copies per reaction.

qPCR reactions were carried out using the GoTaq® qPCR Master Mix (Promega, USA) on DNA (DNA amount ranged from 56 to 106 ng) in a 7500 Real-Time PCR system (Applied Biosystem, USA) with the SybrGreen excitation setting.

Each sample was quantified twice (technical replicate) in a 20 μl reaction volume. The following program was used: 50 °C 2 min; 95 °C 5 min; 40 cycles of (95 °C 30 s, annealing temperature (Table 1) 45 s, 72 °C 1 min); 72 °C 5 min; 95 °C 15 s; 60 °C 1 min; 95 °C 15 s; 60 °C 15 s. Fluorescence was read during the elongation step of each qPCR cycle. An absolute quantification was realized based on the standard curve generated by the plasmid dilutions. The copy number of each sample was normalized to the weight of soil used for DNA extraction to take into account the difference in soil DNA abundance among treatments. qPCR efficiencies were calculated using the formula E = 10^(−1/slope)^ − 1, (where slope is the slope of the standard curve), with E = 1 corresponding to 100% efficiency.

Each qPCR reaction result was subjected to ANOVA followed by a pairwise Tukey’s test. We ran an NMDS ordination based on soil characteristics, using qPCR reaction results and gas fluxes as correlating parameters. Spearman correlations were generated between gene amounts, gas fluxes and soil characteristics. We only present significant correlations (p < 0.05).

## Results

### Soil characteristics

Our soil texture analysis revealed similar clay contents among all three studied areas (Table 2). However, we did identify some differences in soil characteristics among treatments, especially lower soil pH in area YE than NF, and higher NO_3_^−^ content in areas YE than OE compared to NF. Area YE exhibited higher intra-sample heterogeneity than OE and NF. Despite high spatial variability, the low levels of available phosphorus in soil under native vegetation (area NF) reveal the importance of fertilization to achieve better wood production in high weathering soils. Fertilization resulted in considerable differences in the N:P soil ratio.

**Table 2:** Soil characteristics of the three treatment areas: young Eucalyptus plantation (YE), old Eucalyptus plantation (OE), and native forest (NF).

Soil characteristics	YE	OE	NF
Clay content (g.kg^−1^)	600 (14)	592 (23)	600 (20)
Humidity factor (%)	32.8 (3.78) a	24.6 (1.49) b	24.5 (2.86) b
pH (water)	4.00 (0.23) a	4.26 (0.05) ab	4.3 (0.15) b
Total carbon (%)	3.77 (1.03)	3.81 (0.67)	3.02 (0.90)
Total nitrogen (%)	0.22 (0.04)	0.21 (0.04)	0.22 (0.04)
C:N ratio	16.9 (1.65) a	17.5 (0.76) a	13.3 (1.62) b
Available P (mg.kg^−1^)	19 (23)	7 (1)	3 (1)
N:P ratio	221 (109) a	312 (86.7) a	663 (98.7) b
NO_3_^−^ (ugN.g^−1^ of dry soil)	44.6 (14.6) a	3.7 (1.27) b	4.4 (1.3) b
NH_4_^+^ (ugN.g^−1^ of dry soil)	37.9 (16.1)	27.7 (2.89)	26.4 (2.86)
H^+^ + Al^3+^ (cmolc.dm^−3^)	16.3 (2.2)	16 (1.1)	14.5 (1.8)
Ca^2+^ (cmolc.dm^−3^)	0.95 (0.47) a	0.38 (0.07) b	0.31 (0.12) b
Mg^2+^ (cmolc.dm^−3^)	0.29 (0.1) a	0.14 (0.01) b	0.28 (0.04) a
Al^3+^ (cmolc.dm^−3^)	1.86 (0.41)	2.06 (0.22)	1.57 (0.26)

Total C and N are expressed as % weight. Values represent means (n = 5, except n = 4 for humidity, NO_3_^−^ and NH_4_^+^), followed by the standard deviation inside brackets. Different letters mean significant differences among treatments according to Tukey’s test (p < 0.05).

### Gas fluxes

All three treatment areas exhibited similar GHG flux dynamics (Table 3) over the short experimental period. All three areas acted as a sink for CH_4_, with average fluxes ranging from −22 to −35 μg m^−2^ h^−1^. All three areas acted as sources of N_2_O, with average fluxes ranging from 4.8 to 9.4 μg m^−2^ h^−1^. These fluxes did not differ significantly among treatments.

**Table 3:** Gas fluxes in the three treatment areas: young Eucalyptus (YE), old Eucalyptus (OE), and native forest (NF).

Gas flux	YE	OE	NF
CH_4_ (μg CH_4_ m^−2^ h^−1^)	−22.3 (7.3)	−25.2 (10.9)	−34.8 (18.1)
N_2_O (μg N_2_O m^−2^ h^−1^)	9.4 (4.8)	4.8 (3.6)	5.3 (2.7)

Values represent means (n = 5), followed by the standard deviation in brackets. No significant differences were found among treatments according to Tukey’s test (p < 0.05).

### Impact of land use change and *Eucalyptus* logging on microbial community structure

We assessed a total of 525,420 sequences (35,028 per sample) after applying quality filters and data normalization, clustered into 6,831 OTUs (3% dissimilarity threshold). Rarefaction curves show that our sequencing effort describes well the diversity of each sample (Supplemental Fig. 1). Bacterial richness (represented by numbers of OTUs) was significantly different among treatments, being highest in YE, followed by OE, and lastly NF (Table 4). Shannon diversity indices also differed significantly among treatments, being higher in YE and OE than in NF (Table 4). No significant difference was found between the two *Eucalyptus* plantations.

**Table 4:** Richness and diversity index values for the soil bacterial community in the three treatment areas: young Eucalyptus (YE), old Eucalyptus (OE), and native forest (NF).

Richness and diversity index	YE	OE	NF
Number of OTUs	2039 (308) a	1933 (156) ab	1623 (182) b
Shannon index	5.67 (0.18) a	5.54 (0.19) a	4.94 (0.18) b

Values represent means (n = 5), followed by the standard deviation in brackets. Different letters mean significant differences among treatments according to Tukey’s test (p < 0.05).

**Figure 1:**
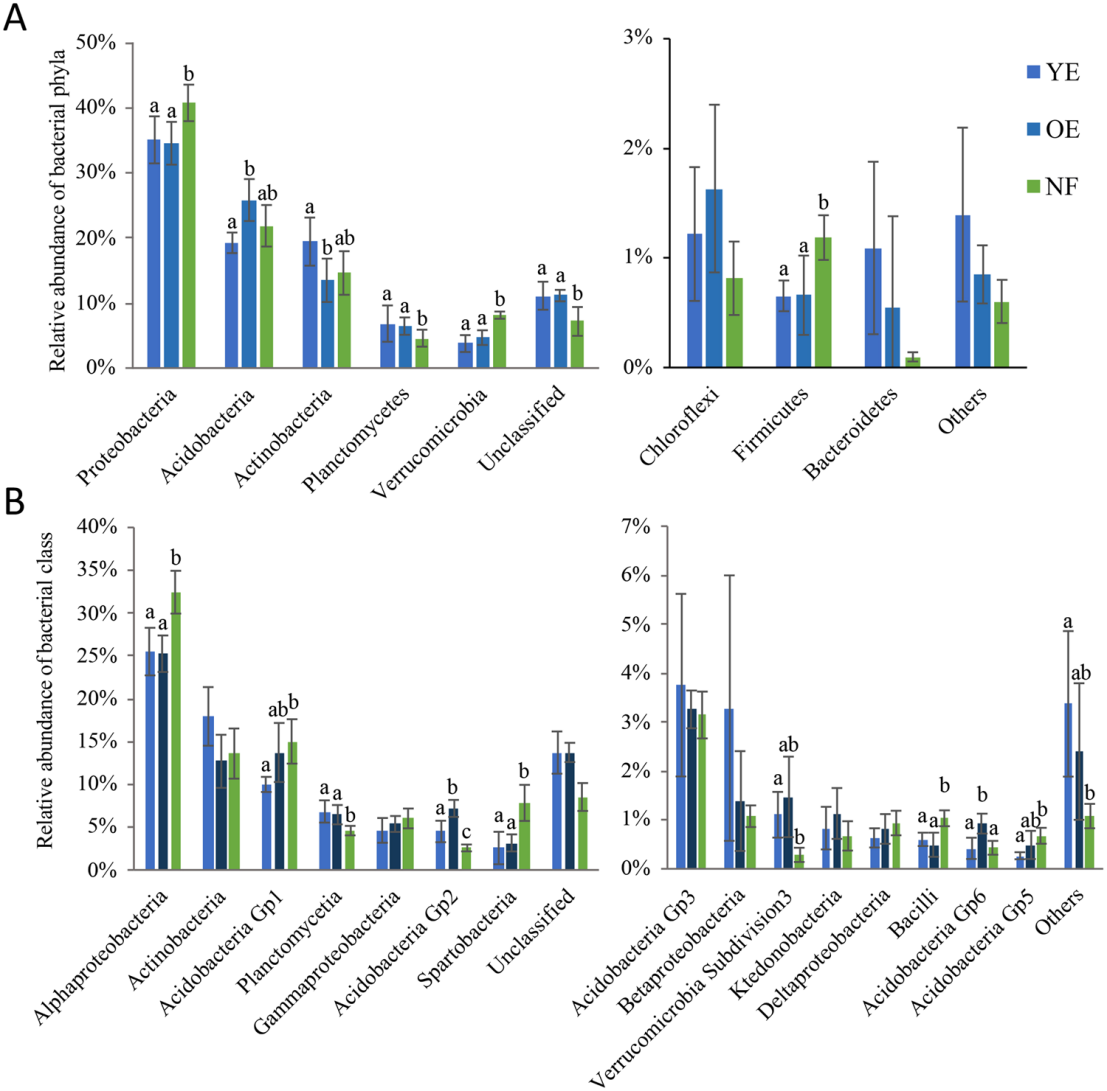
Relative abundances of bacterial phyla (**A**) and classes (**B**) found in soils under Young Eucalyptus (YE), Old Eucalyptus (OE) and Native forest (NF). Taxonomic assignment was based on the RDP database, with an 80% bootstrap threshold. Different letters mean significant differences among treatments according to Tukey’s test (p < 0.05).

Taxonomic assignments revealed that all treatment areas were dominated by the same phyla, but with significant differences in the abundances of some phyla among treatments (Fig. 1A). Eight phyla were found in all treatments: Proteobacteria, Acidobacteria, Actinobacteria, Verrucomicrobia, Chloroflexi, Firmicutes and Bacteroidetes. The three most dominant phyla (Proteobacteria, Acidobacteria and Actinobacteria) represented at least 74% of the communities in each of the three treatments. We could classify approximately 88% of sequences into 16 different classes (Fig. 1B). Alphaproteobacteria were dominant in all treatments, representing 25 to 32% of the entire bacterial community.

Most significant differences in the relative abundances of bacterial taxa were observed between the native forest treatment (NF) and the two *Eucalyptus* plantations (YE and OE). However, there were also significant differences in the microbial community between the YE and OE treatments.

**Figure 2:**
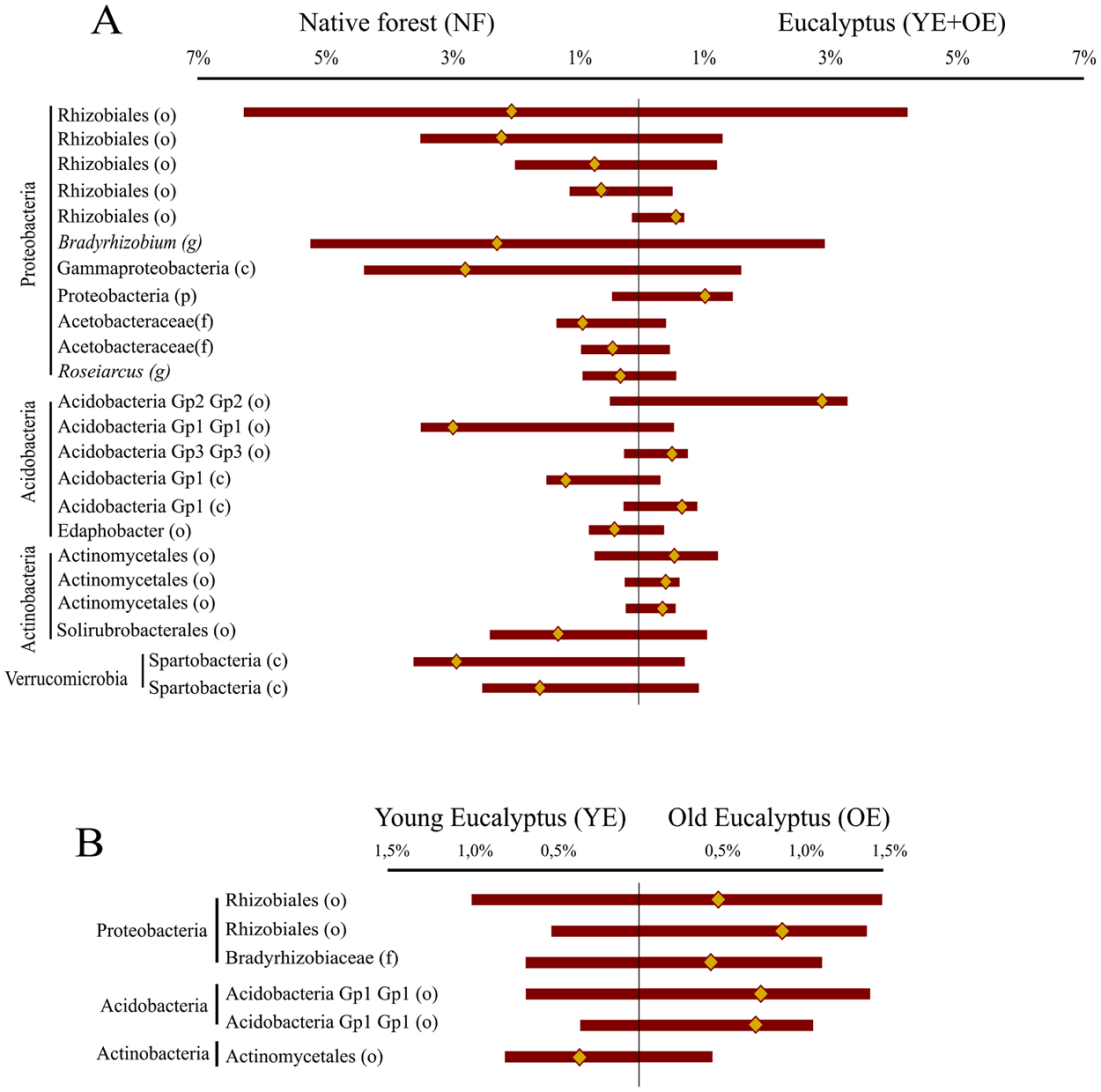
Comparison of the relative abundances of the top 50 OTUs between soils under native forest and Eucalyptus plantations (**A**), and between soils under Young Eucalyptus and Old Eucalyptus. (**B**) Only OTUs with significant differences are represented. Bars represent the average relative abundance of each OTU, extending to the left of the midpoint for one treatment and to the right for the other treatment. The yellow diamonds represent the differences in abundance between the treatments. In the left, the identification of each OTU. o - order, g - genus, f - family, c – class.

A global analysis of the distribution of the 6,831 OTUs indicated that, overall, microbial community structure was significantly affected by treatments, with all treatments differing from one another (PERMANOVA, p < 0.001). To identify the main bacterial groups responsible for the structure change, the abundances of the 50 most abundant OTUs (representing 56% of the entire community) were tested by a ISA, which allowed us to identify 23 OTUs that were significantly different between the YE + OE and NF treatments. The 23 OTUs identification and their relative abundances in each treatment were shown in Fig. 2A. Additionally, the ISA performed on the 50 most abundant OTUs present in both *Eucalyptus* treatments revealed only six OTUs that were significantly different between them. The six OTUs identification and their relative abundances are shown in the Fig. 2B.

### Quantification of abundances of C and N cycle functional genes

**Figure 3:**
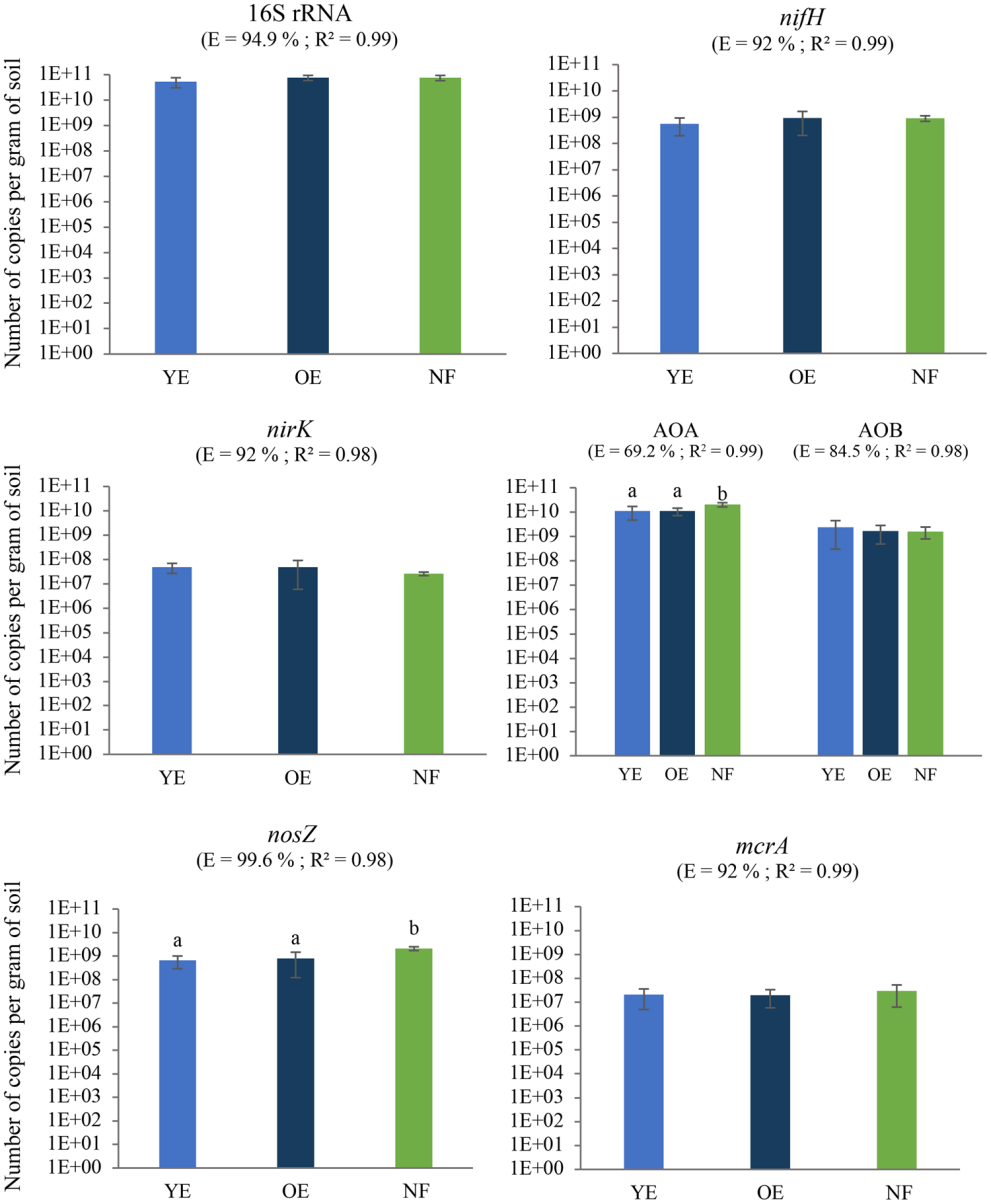
Quantification of 16S rRNA and nitrogen and carbon cycle functional gene abundance in soils under Young Eucalyptus (YE), Old Eucalyptus (OE) and Native forest (NF). The average copy number per gram of soil is plotted (logarithmic scale) for each condition (n = 5). Error bars represent standard deviation. AOA = archaeal amoA; AOB = bacterial amoA. E = significant reaction efficiency. Different letters over the bars represent significant differences according to ANOVA followed by Tukey’s test (p < 0.05).

Our qPCRs were efficient in terms of quantifying functional gene copy numbers (Fig. 3). Dissociation curves indicated that the reactions were specific for all genes (data not shown), with R-squared values of the standard curves ranging from 0.98 to 0.99.

Copy numbers of the 16S rRNA gene were very similar among treatments (approx. 10^10^ copies). No significant differences were observed among treatments for the genes *nifH* (approx. 10^9^ copies), *nirK* (approx. 10^7^ copies), bacterial *amoA* (approx. 10^9^ copies), or *mcrA* (approx. 10^7^ copies). However, copy numbers of the genes *nosZ* and archaeal *amoA* were significantly affected by land use, with both being lower in the two *Eucalyptus* plantations than in native forest. There were no significant differences between YE and OE for these two genes. Archaeal *amoA*, bacterial *amoA*, *nifH* and *nosZ* (approx. 10^9^ to 10^10^ copies) were more abundant compared to *nirK* and *mcrA* (approx. 10^7^ to 10^8^ copies).

### Correlations between abundances of N cycle genes, microbial community composition, soil characteristics, and gas fluxes

To more clearly understand how microbial activities are related to soil physicochemical characteristics, we generated correlations between abundances of N cycle genes (quantified by qPCR) and soil characteristics using Spearman correlations. Three genes exhibited correlations with soil characteristics—*nifH*, archaeal *amoA* and *nosZ* (Table 5)—all of which were correlated with the N:P ratio and available P, whereas archaeal *amoA* and *nosZ* were both also correlated with humidity, NH_4_^+^ and the C:N ratio. We also employed Spearman correlations to investigate the factors linked with gas fluxes in the three treatments, but there were no correlations between N_2_O and CH4 fluxes and N cycle gene abundances or any soil characteristic.

**Table 5:** Spearman correlations (n = 15) between N cycle gene abundances (measured by qPCR) and soil characteristics.

Soil parameter	*nifH*	Archaeal *amoA*	*nosZ*
Humidity	NS	p = 0.01 R = −0.62	p = 0.007 R = −0.66
C:N ratio	NS	p = 0.003 R = −0.71	p = 0.002 R = −0.74
NH_4_^+^	NS	p = 0.01 R = −0.62	p = 0.007 R = −0.66
Available P	p = 0.01 R = −0.61	p = 0.001 R = −0.71	p = 0.001 R = −0.74
N:P ratio	p = 0.04 R = 0.53	p = 0.01 R = 0.64	p = 0.01 R = 0.64

Spearman correlations with p-values < 0.05 are shown. NS means lack of significant correlation. Genes and soil characteristics not shown in this table lacked any correlation. Correlations with ion abundances (K^+^, Na^+^, H^+^ + Al^3+^, Ca, Mg and Al) were not tested.

**Figure 4:**
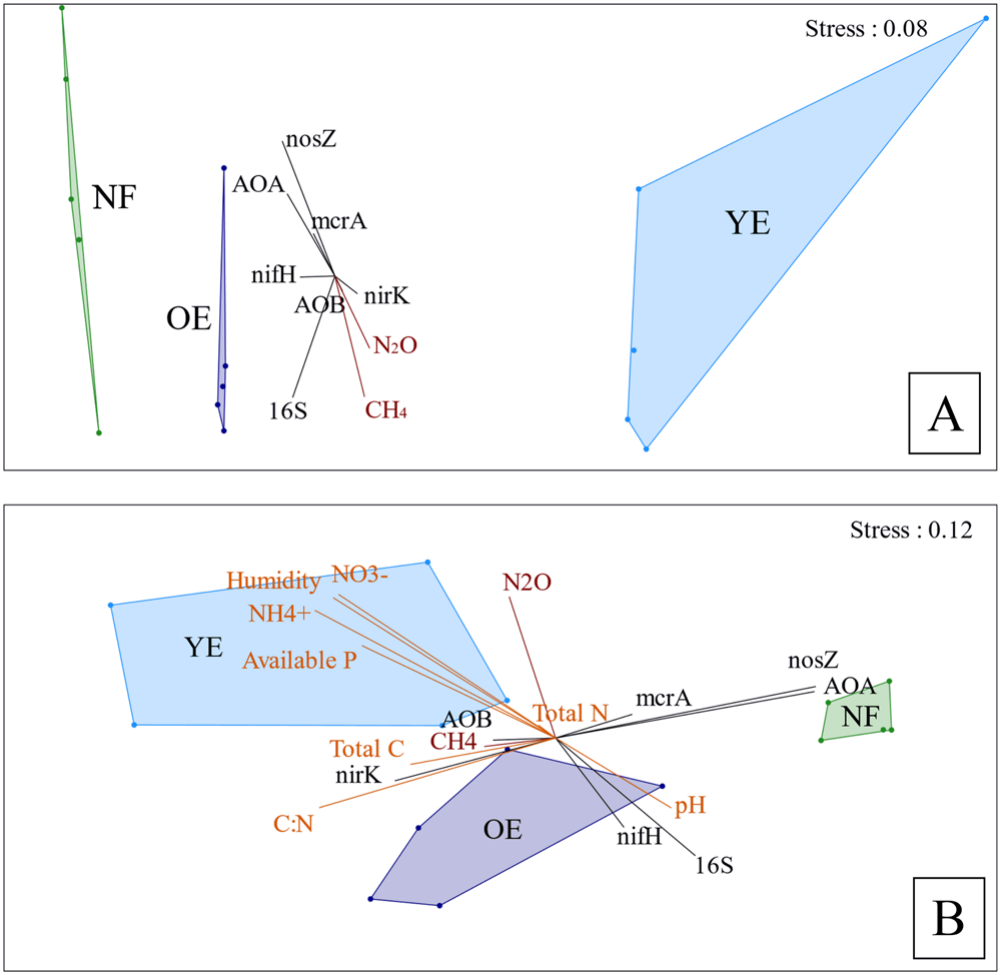
Non-metric multidimensional scaling (NMDS) ordination using Bray-Curtis similarity index of soil characteristics (**A**) and OTU distribution (**B**) for soils under Young Eucalyptus (YE), Old Eucalyptus (OE), and Native forest (NF). Environmental factors are plotted as vectors. AOA = archaeal *amoA*; AOB = bacterial *amoA*. Genes (abundances) are shown in black, gas flux data in red, and soil characteristics are in orange. Stress is expressed in a scale ranging from 0 to 1.

We ran non-metric ordinations (NMDS) to better visualize the data structure and differential relationships in terms of microbial community composition (OTUs) and functional gene abundance (qPCR). The ordination based on soil characteristics revealed that all treatments differed from each other and that the difference between the YE and OE treatments was more pronounced than the difference between the OE and NF treatments (Fig. 4A). This ordination also revealed higher variability within the YE treatment. An ordination based on the distribution of OTUs (Fig. 4B) indicated that differences between the NF and YE + OE treatments were stronger than those between YE and OE. These differences were correlated with gene copy numbers of archaeal *amoA* and *nosZ* (both of which were higher in the NF treatment) and with gene copy number of *nirK*, total C content and the C:N ratio (higher in the two *Eucalyptus* treatments). Moreover, the higher graphical dispersion for the YE and OE treatments compared to the NF treatment on the NMDS ordination indicates higher beta diversity in the *Eucalyptus* treatments than in the NF treatment.

## Discussion

The main phyla we found in the soil samples of the three treatments are common in soils, either from cultivated or native forests^29–33^. A dominance of Proteobacteria has also previously been reported for many types of soils^30,31,33^. Despite the apparent homogeneity in microbial composition across treatments, the PERMANOVA on OTUs indicated that microbial community structure is significantly affected by both land use change (from native forest to *Eucalyptus*) and at the start of a new *Eucalyptus* rotation (the transition from the OE to YE treatment). Conversion of native forest to other land uses—such as silviculture, agriculture or pasture—has previously been shown to have an impact on global soil microbial community structure^34,35^, but we are the first to report a short-term impact (within one month) of *Eucalyptus* plantation logging.

Our results suggest that the main driver of the observed differences in microbial community structure is land use type, as opposed to management practices (i.e., plantation logging and initiation of new rotations) or soil characteristics. Indeed, our NMDS analyses showed that OE and NF treatments were more similar to each other in terms of soil characteristics (Fig. 4A) relative to the YE treatment, but that the microbial community structure of OE was more similar to that of the YE treatment than the NF treatment, meaning that the overlying plant coverage was more effective than soil characteristics in driving microbial community structure (Fig. 4B). A similar outcome has been reported in other studies^36–38^, which indicate that this scenario is more likely to occur when soil pH varies little between treatments, as found in this study. Moreover, our comparison of relative abundances of specific OTUs between the YE + OE treatments and the NF treatment (Fig. 2A) evidence that *Eucalyptus* plantations considerably influence the soil microbial community, including for specific groups of bacteria. High level selection of specific fungal groups by *Eucalyptus* trees has previously been reported^39^.

The higher bacterial alpha diversity (Table 4) and beta diversity (Fig. 4B) of the two *Eucalyptus* treatments compared to the native forest is unexpected considering the greater tree biodiversity of the latter treatment. Interestingly, increased alpha diversity was also reported for an Amazon forest zone 4 months after deforestation^35^. We postulate that microbial richness might increase under these circumstances to adapt to the disturbance caused by deforestation, which is supported by the higher diversity of the YE area in our study given that its tree coverage was removed one month before soil sampling.

However, the higher microbial diversity in the OE area cannot be linked to short-term disturbance, since the trees had been growing there for 6 years before sampling. Higher primary productivity in *Eucalyptus* plantations compared to native forest might explain the higher microbial diversity under *Eucalyptus*, perhaps leading to higher fluxes of root exudates into the soil. The higher soil beta diversity under *Eucalyptus* plantations relative to native forest might be explained by heterogeneous perturbations due to management practices, such as fertilization and the physical consequences of tree-cutting and -dragging before starting a new rotation.

The phyla we identified as being impacted by the three treatments (Proteobacteria, Acidobacteria, Actinobacteria, Planctomycetes, Verrucomicrobia; Fig. 1A) have previously been highlighted as being affected by altered land use, i.e., from forest cover to deforested^34,35,40^. The decreased relative abundance of Proteobacteria we observed between native forest and *Eucalyptus* plantations is consistent with other studies. A similar outcome was observed 20 to 30 years after native forest had been converted to oil palm plantation^34^, and also 2 to 3 years after an Amazonian forest soil lost its forest coverage^40^. Interestingly, both Acidobacteria and Actinobacteria were affected by starting a new *Eucalyptus* rotation (YE vs OE) but not by altered land use (NF vs YE + OE). This result suggests that plantation management practices may have their own inherent impact on microbial community structure.

We found the relative abundance of Acidobacteria to be significantly reduced in the YE treatment compared to that of the OE area. This phylum has previously been shown to be affected by deforestation^40^. Although relative abundances of Acidobacteria (particularly subdivision 1) are considered to be mainly driven by pH^41^, our results suggest that other factors can influence Acidobacteria abundance, as we did not find pH to be significantly correlated with Acidobacteria abundance (data not shown).

The homogenous distribution of 16S rRNA gene sequence abundances among treatments demonstrates that the size of the prokaryote population is not significantly different among treatments. Moreover, the abundance of 16S rRNA gene fragments in our study is within the range usually measured in soil^42^. The higher archaeal *amoA* gene copy number compared to bacterial *amoA* could be linked to the acidic soil pH (<4.5 in all treatments)^43^. This phenomenon has been widely observed in previous soil studies, and may be explained by the fact that NH_3_ content is reduced at lower pH and ammonia-oxidizing Archaea have a higher affinity for NH_3_ than ammonia-oxidizing bacteria^18^.

Since significant differences in gene abundances were only observed between native forest and *Eucalyptus* treatments, and not between the *Eucalyptus* treatments themselves, it seems that land use change and not starting a new rotation has an impact on microbial processes linked to the N and C cycles. Only the archaeal *amoA* and *nosZ* genes were significantly more abundant in soils under native forest than those under *Eucalyptus*. These differences are likely due to the specific soil characteristics of each treatment regime, since both archaeal *amoA* and *nosZ* gene abundances were strongly correlated with certain soil characteristics (Table 5).

The negative correlation between archaeal *amoA* abundance and humidity might be due to the aerobic character of ammonia-oxidizing Archaea, even though these bacteria have been previously shown to tolerate a broad range of oxygen concentrations^44^. The negative correlation between the C:N ratio and archaeal *amoA* abundance seems logical, given that a lower C:N ratio means higher N abundance, so there is more substrate for ammonia-oxidizing Archaea^45^. However, a negative correlation was observed between NH_4_^+^ and archaeal *amoA* abundance, suggesting that higher NH_4_^+^ levels are more a consequence of low nitrifier activity than a cause of higher rates of nitrification^46^.

A similar process has been suggested to explain a negative correlation between soil NO_3_^−^ levels and *nirK* gene abundance^38^. However, the correlations we report between levels of NH_4_^+^ and NO_3_^−^ and humidity should be viewed with caution, as these factors were not measured specifically at each soil-sampling site, but at four sites randomly distributed in the three treatment areas. Thus, the correlations are based on average values for each treatment area, which could lead to spurious correlations.

The negative correlation between *nosZ* gene copy number and humidity is unexpected, because nitrous oxide oxidase has been reported to be highly sensitive to O_2_, even being inhibited by low oxygen levels^47^. However, another study found a higher gene copy number of *nosZ* in wetlands than dry areas^48^, and a negative correlation between levels of *nosZ* and NH_4_^+^ has been observed in wetlands^48^. This correlation might mean that lower NH_4_^+^ levels are a consequence of higher NH_4_^+^ oxidation activity from high levels of nitrous oxide reductase. It is important to note that we measured gene copy number, and not gene expression, in this study. Gene copy number is not always an effective means of reporting microbial activity as it provides no information about gene expression or protein activity.

The negative correlation we revealed between available P and the *nifH*, archaeal *amoA* and *nosZ* genes suggests an important link between P availability and N cycle functioning. However, this finding is not consistent with previous data, which suggest increased P availability promotes nitrification, denitrification and nitrogen fixation^49^. Analysing the bacterial community structure (Fig. 4) we can speculate that this correlation is more likely to be occurring indirectly. The microbial community structure of YE (highly disturbed) is positively correlated with P availability and negatively correlated with *nifH* and *nosZ* and *AOA*. Therefore, the disturbance could be the main factor controlling these correlations.

The absence of a correlation between levels of functional genes and gas fluxes is surprising because correlations between the *nosZ*, archaeal *amoA*, and bacterial *amoA* genes and N_2_O fluxes have previously been described in forest systems^16–18^. The quantification results for *pmoA* may explain CH_4_ fluxes, as this gene has been shown to be positively correlated to CH_4_ emissions from soil^17^. Moreover, we only conducted gene quantification for a single time-period, so we cannot rule out a correlation between gene abundances and gas fluxes over time. Despite the absence of an overall link between microbial structure and functional gene abundance and gas fluxes, it is important to note that our study focused solely on bacterial activities (apart from the archaeal *amoA* gene). It would be interesting to also analyse Archaea and fungi, both of which play important roles in N cycling in soils, especially in forest soils^50^.

In conclusion, we demonstrate that soils under *Eucalyptus* plantations can harbour a distinct and more diverse bacterial community compared to soils under native forest and that this community responds very quickly to environmental disturbances, such as implementation of a new plantation rotation. However, the short-term changes we observed arising from plantation management practices were qualitative and not quantitative, and they did not result in significant changes in terms of greenhouse gas fluxes from soil.

## Electronic supplementary material

Supplemental material

## References

[1] Hughes Lesley (2000). Biological consequences of global warming: is the signal already apparent?. Trends in Ecology & Evolution.

[2] World Meteorological organization. The State of Greenhouse Gases in the Atmosphere Base on Global Observations through 2015. *WMO Greenh. Gas Bull*. 1–8 (2016).

[3] IPCC. Climate change 2007: the physical science basis. *Intergov. Panel Clim. Chang*. **446**, 727–8 (2007).

[4] IPCC. *Climate Change 2014: Mitigation of Climate Change*. *Working Group III Contribution to the Fifth Assessment Report of the Intergovernmental Panel on Climate Change*, 10.1017/CBO9781107415416 (2014).

[5] Matthews HD (2014). National contributions to observed global warming. Environ. Res. Lett..

[6] Brazilian tree industry. *Annual report of IBA (industria brasileira de árvores)*. (2016).

[7] Forestry Department of the FAO. *Forest ResourcesAssessment 2010 Country Report - Brazil* (2010).

[8] Burrows WH (2002). Growth and carbon stock change in eucalypt woodlands in northeast Australia: ecological and greenhouse sink implications. Glob. Chang. Biol..

[9] Du H (2015). Carbon Storage in a Eucalyptus Plantation Chronosequence in Southern China. Forests.

[10] Zhang K (2017). Impact of nitrogen fertilization on soil-Atmosphere greenhouse gas exchanges in eucalypt plantations with different soil characteristics in southern China. PLoS One.

[11] Kumar, N., Meghabarot, J., Gupta, P. & Patel, K. An Evaluation of Short Term Greenhouse Gas Emissions from Soil and Atmosphere Exchange in Response to Controlling Edaphic Factgors of Eucalyptus Plantation, Gujarat, India. *Int. J. Environ*. **3** (2014).

[12] Oertel C, Matschullat J, Zurba K, Zimmermann F, Erasmi S (2016). Greenhouse gas emissions from soils—A review. Chemie der Erde - Geochemistry.

[13] Rousk, J. & Bengtson, P. Microbial regulation of global biogeochemical cycles. *Front. Microbiol*. **5** (2014). 10.3389/fmicb.2014.00103 PMC3954078 24672519

[14] Insam H, Wett B (2008). Control of GHG emission at the microbial community level. Waste Manag..

[15] Bardgett RD, Freeman C, Ostle NJ (2008). Microbial contributions to climate change through carbon cycle feedbacks. ISME J..

[16] Morales SE, Cosart T, Holben WE (2010). Bacterial gene abundances as indicators of greenhouse gas emission in soils. ISME J..

[17] Martins CSC, Nazaries L, Macdonald CA, Anderson IC, Singh BK (2015). Water availability and abundance of microbial groups are key determinants of greenhouse gas fluxes in a dryland forest ecosystem. Soil Biol. Biochem..

[18] Wang Y (2016). Relationships between ammonia-oxidizing communities, soil methane uptake and nitrous oxide fluxes in a subtropical plantation soil with nitrogen enrichment. Eur. J. Soil Biol..

[19] Alves BJR (2012). Selection of the most suitable sampling time for static chambers for the estimation of daily mean N2O flux from soils. Soil Biol. Biochem..

[20] Embrapa S. *Manual de Métodos de Análise de Solo*. (Embrapa Solos, 2011).

[21] de Morais RF, Boddey RM, Urquiaga S, Jantalia CP, Alves BJR (2013). Ammonia volatilization and nitrous oxide emissions during soil preparation and N fertilization of elephant grass (Pennisetum purpureum Schum.). Soil Biol. Biochem..

[22] Apprill A, McNally S, Parsons R, Weber L (2015). Minor revision to V4 region SSU rRNA 806R gene primer greatly increases detection of SAR11 bacterioplankton. Aquat. Microb. Ecol..

[23] Parada AE, Needham DM, Fuhrman JA (2016). Every base matters: assessing small subunit rRNA primers for marine microbiomes with mock communities, time series and global field samples. Environ. Microbiol..

[24] Schloss PD (2009). Introducing mothur: Open-Source, Platform-Independent, Community-Supported Software for Describing and Comparing Microbial Communities. Appl. Environ. Microbiol..

[25] Quast C (2013). The SILVA ribosomal RNA gene database project: improved data processing and web-based tools. Nucleic Acids Res..

[26] Cole JR (2009). The Ribosomal Database Project: improved alignments and new tools for rRNA analysis. Nucleic Acids Res..

[27] Hammer R, Harper DAT, Ryan PD (2001). PAST: Paleontological Statistics Software Package for Education and Data Analysis–Palaeontol. Electron..

[28] Dufrene M, Legendre P (1997). Species assemblages and indicator species: The need for a flexible asymmetrical approach. Ecol. Monogr..

[29] Rachid CTCC (2013). Effect of sugarcane burning or green harvest methods on the Brazilian Cerrado soil bacterial community structure. PLoS One.

[30] Castañeda LE, Barbosa O (2017). Metagenomic analysis exploring taxonomic and functional diversity of soil microbial communities in Chilean vineyards and surrounding native forests. PeerJ.

[31] Quirino BF (2009). Molecular phylogenetic diversity of bacteria associated with soil of the savanna-like Cerrado vegetation. Microbiol Res..

[32] Araujo JF (2012). Characterization of soil bacterial assemblies in brazilian savanna-like vegetation reveals acidobacteria dominance. Microb. Ecol..

[33] Siles JA, Rachid CTCC, Sampedro I, García-Romera I, Tiedje JM (2014). Microbial Diversity of a Mediterranean Soil and Its Changes after Biotransformed Dry Olive Residue Amendment. PLoS One.

[34] Tripathi BM (2016). The impact of tropical forest logging and oil palm agriculture on the soil microbiome. Mol. Ecol..

[35] Navarrete AA (2015). Soil microbiome responses to the short-term effects of Amazonian deforestation. Mol. Ecol..

[36] Lauber CL, Ramirez KS, Aanderud Z, Lennon J, Fierer N (2013). Temporal variability in soil microbial communities across land-use types. ISME J..

[37] Leff JW (2015). Consistent responses of soil microbial communities to elevated nutrient inputs in grasslands across the globe. Proc. Natl. Acad. Sci..

[38] Rachid C.T.C.C., Balieiro F.C., Peixoto R.S., Pinheiro Y.A.S., Piccolo M.C., Chaer G.M., Rosado A.S. (2013). Mixed plantations can promote microbial integration and soil nitrate increases with changes in the N cycling genes. Soil Biology and Biochemistry.

[39] Rachid CTCC (2015). Intercropped Silviculture Systems, a Key to Achieving Soil Fungal Community Management in Eucalyptus Plantations. PLoS One.

[40] Mendes LW (2015). Soil-Borne Microbiome: Linking Diversity to Function. Microb. Ecol..

[41] Sait M, Davis KER, Janssen PH (2006). Effect of pH on Isolation and Distribution of Members of Subdivision 1 of the Phylum Acidobacteria Occurring in Soil. Appl. Environ. Microbiol..

[42] Henry S, Bru D, Stres B, Hallet S, Philippot L (2006). Quantitative Detection of the nosZ Gene, Encoding Nitrous Oxide Reductase, and Comparison of the Abundances of 16S rRNA, narG, nirK, and nosZ Genes in Soils. Appl. Environ. Microbiol..

[43] Prosser JI, Nicol GW (2012). Archaeal and bacterial ammonia-oxidisers in soil: the quest for niche specialisation and differentiation. Trends Microbiol..

[44] Erguder TH, Boon N, Wittebolle L, Marzorati M, Verstraete W (2009). Environmental factors shaping the ecological niches of ammonia-oxidizing archaea. FEMS Microbiol. Rev..

[45] Priha O, Smolander A (1999). Nitrogen transformations in soil under Pinus sylvestris, Picea abies and Betula pendula at two forest sites. Soil Biol. Biochem..

[46] Erickson H, Keller M, Davidson EA (2001). Nitrogen Oxide Fluxes and Nitrogen Cycling during Postagricultural Succession and Forest Fertilization in the Humid Tropics. Ecosystems.

[47] Wrage N, Velthof G, van Beusichem M, Oenema O (2001). Role of nitrifier denitrification in the production of nitrous oxide. Soil Biol. Biochem..

[48] Ligi T (2014). Effects of soil chemical characteristics and water regime on denitrification genes (nirS, nirK, and nosZ) abundances in a created riverine wetland complex. Ecol. Eng..

[49] Vance, C. P., Graham, P. H. & Allan, D. L. In *Nitrogen Fixation: From Molecules to Crop Productivity* 509–514 (Kluwer Academic Publishers, 10.1007/0-306-47615-0_291 (2000).

[50] Waring BG, Averill C, Hawkes CV (2013). Differences in fungal and bacterial physiology alter soil carbon and nitrogen cycling: insights from meta-analysis and theoretical models. Ecol. Lett..

[51] Muyzer G, de Wall EC, Uitterlinden AG (1993). Profiling of complex microbial populations by denaturing gradient gel electrophoresis analysis of polymerase chain reaction-amplified genes coding for 16S rRNA. Appl. Environ. Microbiol..

[52] Barlaan EA, Sugimori M, Furukawa S, Takeuchi K (2005). Electronic microarray analysis of 16S rDNA amplicons for bacterial detection. J. Biotechnol..

[53] Poly F, Ranjard L, Nazaret S, Gourbiere F, Monrozier LJ (2001). Comparison of nifH Gene Pools in Soils and Soil Microenvironments with Contrasting Properties. Appl. Environ. Microbiol..

[54] Leininger S (2006). Archaea predominate among ammonia-oxidizing prokaryotes in soils. Nature.

[55] Le Roux X (2008). Effects of aboveground grazing on coupling among nitrifier activity, abundance and community structure. ISME J..

[56] Rotthauwe, J. -H., Witzel, K. -P. & Liesack, W. The Ammonia Monooxygenase Structural Gene amoA as a Functional Marker: Molecular Fine-Scale Analysis of Natural Ammonia-Oxidizing Populations. *Appl. Environ. Microbiol*. 4704–4712 (1997). 10.1128/aem.63.12.4704-4712.1997 PMC168793 9406389

[57] Hallin S, Lindgren P (1999). PCR Detection of Genes Encoding Nitrite Reductase in Denitrifying Bacteria. Society.

[58] Throbäck IN, Enwall K, Jarvis A, Hallin S (2004). Reassessing PCR primers targeting nirS, nirK and nosZ genes for community surveys of denitrifying bacteria with DGGE. FEMS Microbiol. Ecol..

[59] Denman SE, Tomkins NW, McSweeney CS (2007). Quantitation and diversity analysis of ruminal methanogenic populations in response to the antimethanogenic compound bromochloromethane. FEMS Microbiol. Ecol..

[60] Anantasook N, Wanapat M, Cherdthong A, Gunun P (2013). Changes of Microbial Population in the Rumen of Dairy Steers as Influenced by Plant Containing Tannins and Saponins and Roughage to Concentrate Ratio. Asian-Australasian J. Anim. Sci..

