# Supplementary material for: Short-term effect of Eucalyptus plantations on soil microbial communities and soil-atmosphere methane and nitrous oxide exchange
Source: Sci Rep. 2018 Oct 11;8:15133. doi: 10.1038/s41598-018-33594-6 (PMC6181980; doi:10.1038/s41598-018-33594-6)
Supplement: Supplementary file 1 — Supplemental material [file 41598_2018_33594_MOESM1_ESM.docx]

**Short-term effect of *Eucalyptus* plantations on soil microbial communities and soil-atmosphere methane and nitrous oxide exchange**

Caroline Cuer, Renato de A R Rodrigues, Fabiano C Balieiro, Jacqueline Jesus, Elderson Silva, Bruno José R Alves, Caio T C C Rachid


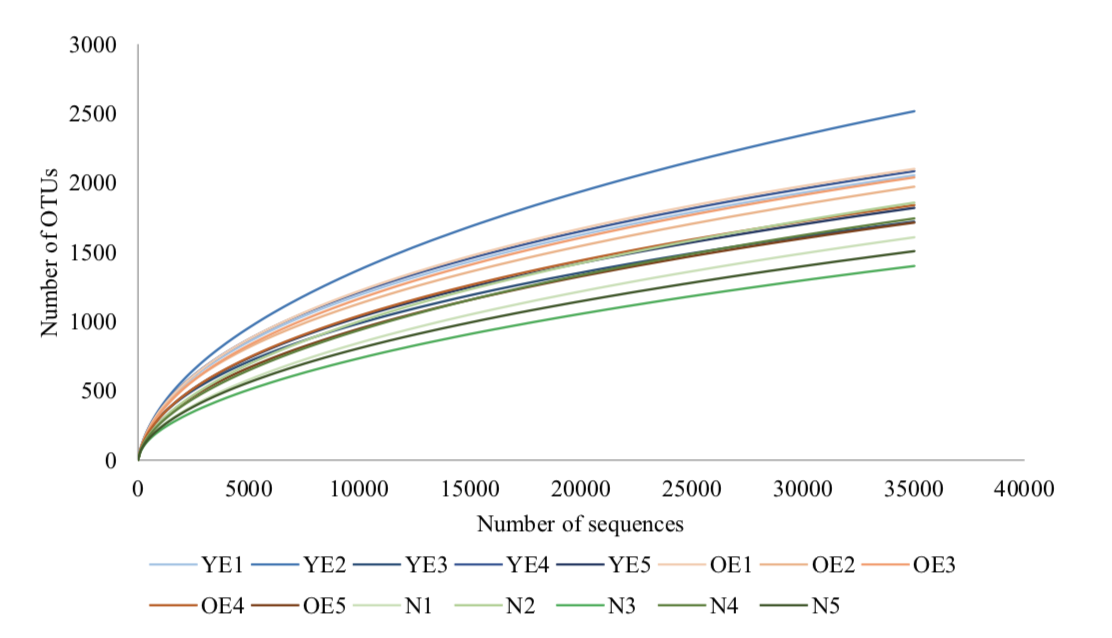


Supplementary figure 1. Rarefaction curves per sample. YE (young Eucalyptus plantation) in blue shades, OE (old Eucalyptus plantation) in orange shades and N (native forest) in green shades.

Supplemental Table 1 - Full qPCR standard curve descriptors

| Target gene | Eficiency | Slope | Intercept | no template controls |
| --- | --- | --- | --- | --- |
| nifH | 92% | -3.531 | 43.34 | not detected |
| nirK | 92% | -3.536 | 36.82 | not detected |
| mcrA | 92% | -3.537 | 46.54 | not detected |
| nosZ | 99% | -3.332 | 44.69 | not detected |
| 16S | 95% | -3.45 | 43.177 | not detected |
| AOA | 69% | -4.374 | 54.61 | not detected |
| AOB | 84% | -3.757 | 53.448 | not detected |
